# Association between fasting blood glucose levels at admission and overall survival of patients with pancreatic cancer

**DOI:** 10.1186/s12885-021-07859-9

**Published:** 2021-02-06

**Authors:** Mingming Zhang, Xiaoru Hu, Ye Kang, Wanfeng Xu, Xianghong Yang

**Affiliations:** 1 Department of Pathology, Shengjing Hospital of China Medical University, Shenyang, China; 2 Department of Endocrinology, Shengjing Hospital of China Medical University, Shenyang, China

**Keywords:** Pancreatic cancer, Blood glucose, Survival, Prognosis

## Abstract

**Background:**

The associations between fasting blood glucose and staging and overall survival of patients with pancreatic cancer are still controversial. This study aimed to investigate the association between fasting blood glucose levels and overall survival (OS) of patients with pancreatic cancer and to evaluate the impact of differentiation and staging of pancreatic cancer.

**Methods:**

This was a retrospective study of patients with pathologically confirmed pancreatic cancer admitted to Shengjing Hospital of China Medical University between 01/2012 and 12/2016. The outcome was the OS. The factors associated with OS were examined using univariable and multivariable Cox and logistic regression analyses.

**Results:**

A total of 253 patients were included. Preoperative blood glucose levels were not significantly associated with the OS of patients with pancreatic cancer (HR = 1.04, 95%CI: 0.78–1.40, *P* = 0.781). Only CA199 > 1000 was independently associated with OS (HR = 1.86, 95%CI: 1.15–3.02, *P* = 0.012). The median survival in the normal glucose group was 20.5 months (95% confidence interval (CI): 14.2–26.9). The median survival in the high glucose group was 14.2 months (95% CI: 9.7–18.6). There was no statistically significant difference between the two groups (*P* = 0.573). Multivariable logistic regression analyses were performed to determine if blood glucose levels influenced the 1- and 2-year OS. No significant association was observed for 1-year (OR = 1.27, 95%CI: 0.71–2.29, *P* = 0.418) or 2-year (HR = 1.37, 95%CI: 0.76–2.46, *P* = 0.296) OS.

**Conclusions:**

Fasting blood glucose levels are not associated with the OS of patients with pancreatic adenocarcinoma.

## Background

Pancreatic adenocarcinoma (ductal and its variants) is a malignant exocrine tumor that is responsible for > 90% of all pancreatic cancers [1–3]. Most tumors (60–70%) occur in the head of the pancreas [1], and it most commonly affects patients > 55 years old [1–3]. The worldwide age-standardized annual incidence rate of pancreatic cancer is 5.5 per 100,000 men and 4 per 100,000 women [4, 5]. Pancreatic cancer is the tenth most common cancer in men and the ninth most common cancer in women [4, 5]. Major risk factors for pancreatic cancer include tobacco use, genetic predisposition and family history, obesity, chronic pancreatitis, and preexisting diabetes [1–3, 6]. The prognosis of pancreatic adenocarcinoma is very poor, with 5-year overall survival of < 10% [1–3].

Diabetes is both a risk factor and a complication of pancreatic cancer [1–3, 6]. Indeed, prediabetes and long-term diabetes are associated with an increased risk of pancreatic cancer [7, 8], and early-stage pancreatic cancer causes new-onset diabetes, but the pathogenesis is unclear. A systematic review also showed that elevated fasting blood glucose levels are associated with an increased risk of pancreatic cancer [9].

Diabetes is associated with a poor prognosis in patients with different solid tumors, such as head & neck, breast, liver, bladder, colorectal, and endometrial cancer [10–12]. High blood glucose levels are associated with the aggressiveness of colorectal cancer [13]. However, the association of fasting blood glucose with staging and overall survival (OS) of pancreatic cancer patients is still controversial [14, 15], and there are few studies in China or Asia that address this issue. The studies that explored the association between fasting blood glucose levels and the prognosis of pancreatic cancer have had inconsistent conclusions [14, 15]. These differences in results may be because of different populations with different genetic characteristics, cancer stage, or cancer differentiation. However, few studies have considered whether the differentiation and staging of pancreatic cancer influenced the results.

Therefore, the aim of the present study was to investigate the association between fasting blood glucose levels and OS of patients with pancreatic cancer and to evaluate the impact of differentiation and staging of pancreatic cancer on this association.

## Methods

### Study design and patients

This was a retrospective study of patients with pathologically confirmed pancreatic cancer admitted to Shengjing Hospital of China Medical University between January 2012 and December 2016. This study was approved by the Ethics Committee of Shengjing Hospital of China Medical University. Informed consent was waived because of the retrospective design.

The inclusion criteria were 1) > 18 years and 2) pathologically diagnosed with pancreatic cancer. The exclusion criteria were 1) severe primary diseases such as respiratory, cardiovascular, cerebrovascular, liver, kidney, or hematopoietic disease, psychosis, or long-term medication, or 2) incomplete clinical data.

### Diagnostic criteria

The diagnosis and staging were made according to the NCCN guidelines using pancreatic computed tomography (CT) and/or magnetic resonance imaging (MRI) with contrast and a biopsy [6]. Pathological diagnosis and staging were made according to the WHO Classification of Tumours of the Digestive System (4th edition) [16], pancreatic adenocarcinoma included ductal adenocarcinoma and its variants: adenosquamous carcinoma, colloid carcinoma (mucinous non-cystic carcinoma), hepatoid carcinoma, medullary carcinoma, signet-ring carcinoma, undifferentiated carcinoma, and undifferentiated carcinoma with osteoclast-like giant cells.

### Grouping and treatments

The patients were assigned to the normal blood glucose group and the hyperglycemia group according to a fasting blood glucose cutoff of 6.11 mmol/L. All patients were treated according to the current guidelines when they were diagnosed [6].

### Data collection and outcomes

Demographic information (sex and age), current medical history (disease course, diameter of tumor, site of tumor, presence or absence of lymphatic metastasis, AJCC TNM 8 staging, and differentiation), medical history (hypertension, diabetes mellitus, smoking, and drinking), family history, pathological type, and surgical procedure were collected. Mortality was defined as death during the whole follow-up period. Perioperative complications and adverse reactions after surgical treatment were collected. OS was defined as the time from diagnosis to death from any cause.

### Follow-up

Follow-up was conducted routinely by outpatient visits, hospitalization, and/or telephone. The follow-up information included death or survival and was extracted from the medical charts. Follow-up was censored on December 31st, 2019.

### Statistical analysis

SPSS 22.0 (IBM, Armonk, NY, USA) was used for data analysis. The continuous data were first analyzed using the Kolmogorov-Smirnov test to determine whether they were normally distributed. The continuous variables conforming to the normal distribution are expressed as means ± standard deviation and were tested using the two-sample unpaired t-test; otherwise, they are presented as medians (minimum, maximum) and analyzed using the Mann-Whitney U test. Categorical data are presented as n (%) and were analyzed using the chi-square test or Fisher’s exact probability test. Univariable Cox regression was used to analyze the factors affecting OS. Variables with *P* < 0.05 or clinical significance were included in the multivariable Cox regression. Logistic regression was used to determine the variables independently associated with 1- and 2-year survival. Kaplan-Meier survival curves were plotted according to OS under different exposure factors and were compared using the log-rank test. Two-sided *P*-values < 0.05 were considered statistically significant.

## Results

### Characteristics of the patients

During the study period, 312 patients were admitted for pancreatic cancer, but 59 were excluded because of a lack of follow-up at the study hospital or missing blood glucose data. Therefore, 253 patients were included, of whom 208 were deceased, and 45 were alive or lost to follow-up. The demographic data are presented in Table 1.

**Table 1:** Characteristics of the patients

	All (*n* = 253)	Normal blood glucose (*n* = 94)	Hyperglycemia (*n* = 159)	P
Age, years, median (range)	60 (37,83)	60 (37,80)	61 (38,83)	0.516
BMI, median (range)	23.3 (12.4,32.1)	22.3 (15.5,29)	23.5 (12.4,32.1)	0.039
Sex, n (%)				0.002
Female	106 (41.7%)	27 (28.7%)	79 (49.4%)	
Male	148 (58.5%)	67 (71.3%)	81 (50.9%)	
Smoking history, n (%)	93 (36.8%)	46 (48.9%)	47 (29.6%)	0.002
Drinking history, n (%)	49 (19.4%)	21 (22.3%)	28 (17.7%)	0.370
Diabetes history, n (%)	65 (25.7%)	7 (7.4%)	58 (36.5%)	< 0.001
Hypertension history, n (%)	70 (27.7%)	19 (20.2%)	51 (32.1%)	0.042
Allergic history, n (%)	33 (13%)	7 (7.4%)	26 (16.4%)	0.042
Abdominal pain, n (%)	174 (69.9%)	69 (74.2%)	105 (67.3%)	0.252
Back pain, n (%)	38 (15.3%)	17 (18.3%)	21 (13.5%)	0.306
Anorexia, n (%)	10 (4%)	2 (2.2%)	8 (5.1%)	0.329
Loss of appetite, n (%)	106 (42.6%)	37 (39.8%)	69 (44.2%)	0.493
Loss of weight, n (%)	154 (61.8%)	50 (53.8%)	104 (66.7%)	0.043
CA199, n (%)				0.383
Normal	51 (21.9%)	22 (24.7%)	29 (20.1%)	
37–1000	147 (63.1%)	57 (64%)	90 (62.5%)	
> 1000	35 (15%)	10 (11.2%)	25 (17.4%)	
Surgical procedure, n (%)				0.932
Whipple	192 (75.9%)	73 (77.7%)	119 (74.8%)	
Distal	52 (20.6%)	18 (19.1%)	34 (21.4%)	
Other	9 (3.6%)	3 (3.2%)	6 (3.8%)	
Site of onset, n (%)				0.790
Head of pancreas	201 (79.8%)	75 (80.6%)	126 (79.2%)	
Tail of pancreas	51 (20.2%)	18 (19.4%)	33 (20.8%)	
Differentiation, n (%)				0.613
Poor	61 (24.1%)	21 (22.3%)	40 (25.2%)	
Moderate-high	192 (75.9%)	73 (77.7%)	119 (74.8%)	
AJCC8 staging				0.677
Stage I	121 (47.8%)	48 (51.1%)	73 (45.9%)	
Stage II	86 (34%)	31 (33%)	55 (34.6%)	
Stage III-IV	46 (18.2%)	15 (16%)	31 (19.5%)	
Obstructive jaundice	129 (51%)	44 (46.8%)	85 (53.5%)	0.307
Follow-up time, months, median (range)	16.5 (0.1,95)	20.9 (0.5,95)	14.2 (0.1,93.3)	0.327

*BMI* body mass index, *CA* cancer antigen, *AJCC* American Joint Committee on Cancer

Compared with the patients with normal glucose, the hyperglycemia group had a higher body mass index (BMI) (median, 23.5 vs. 22.3 kg/m^2^, *P* = 0.039), and higher proportions of females (49.4% vs. 28.7%, *P* = 0.002), diabetes (36.5% vs. 7.4%, *P* < 0.001), hypertension (32.1% vs. 20.2%, *P* = 0.042), allergies (16.4% vs. 7.4%, *P* = 0.042), and weight loss (66.7% vs. 53.8%, *P* = 0.043). Cancer differentiation degree and AJCC8 stage were not significantly different between the normal glucose and the hyperglycemia groups.

### Factors associated with OS

In the univariable analyses, only CA199 and back pain were associated with OS. Cancer differentiation degree and AJCC8 stage were not identified as associated factors. Blood glucose was included in the Cox multivariable analysis along with CA199 and back pain. The results showed that preoperative blood glucose was not significantly associated with the OS of patients with pancreatic cancer (HR = 1.04, 95%CI: 0.78–1.40, *P* = 0.781). The glucose levels were tested as a continuous variable in the Cox model, and similar results were observed. Only CA199 > 1000 was independently associated with OS (HR = 1.86, 95%CI: 1.15–3.02, *P* = 0.012) (Table 2).

**Table 2:** Univariable and multivariable Cox regression analyses of OS

	Univariable			Multivariable		
	HR	95% CI	P	HR	95% CI	P
Age	1.01	(0.99,1.02)	0.308			
BMI	0.98	(0.94,1.03)	0.418			
Blood glucose						
Normal	ref					
Abnormal	1.08	(0.82,1.44)	0.574	1.04	(0.78,1.4)	0.781
Sex						
Female	ref					
Male	1.07	(0.81,1.41)	0.621			
Smoking history						
No	ref					
Yes	1.14	(0.86,1.51)	0.358			
Drinking history						
No	ref					
Yes	1.30	(0.94,1.81)	0.113			
Diabetes						
No	ref					
Yes	1.05	(0.77,1.43)	0.771			
Hypertension						
No	ref					
Yes	0.94	(0.69,1.27)	0.683			
Allergic history						
No	ref					
Yes	1.22	(0.83,1.78)	0.314			
Abdominal pain						
No	ref					
Yes	1.08	(0.8,1.47)	0.607			
Back pain						
No	ref					
Yes	0.61	(0.4,0.92)	0.019	0.65	(0.42,1.03)	0.064
Anorexia						
No	ref					
Yes	0.86	(0.4,1.82)	0.689			
Loss of appetite						
No	ref					
Yes	1.09	(0.82,1.43)	0.559			
Loss of weight						
No	ref					
Yes	0.82	(0.62,1.09)	0.165			
CA199						
Normal	ref					
37–1000	1.47	(1.02,2.12)	0.039	1.38	(0.94,2.01)	0.099
> 1000	1.84	(1.14,2.97)	0.013	1.86	(1.15,3.02)	0.012
Surgical procedure						
Whipple	ref					
Distal	1.26	(0.91,1.75)	0.161			
Other	0.95	(0.44,2.02)	0.886			
Location						
Head	ref					
Tail	1.21	(0.87,1.69)	0.253			
Differentiation degree						
Poor	ref					
Moderate-high	0.79	(0.57,1.08)	0.144			
AJCC8 staging						
Stage I	ref					
Stage II	1.05	(0.77,1.43)	0.753			
Stage III-IV	1.30	(0.9,1.87)	0.162			
Obstructive jaundice						
No	ref					
Yes	0.89	(0.68,1.17)	0.405			

*HR* hazards ratio, *CI* confidence interval, *BMI* body mass index, *CA* cancer antigen, *AJCC* American Joint Committee on Cancer

**Fig. 1:**
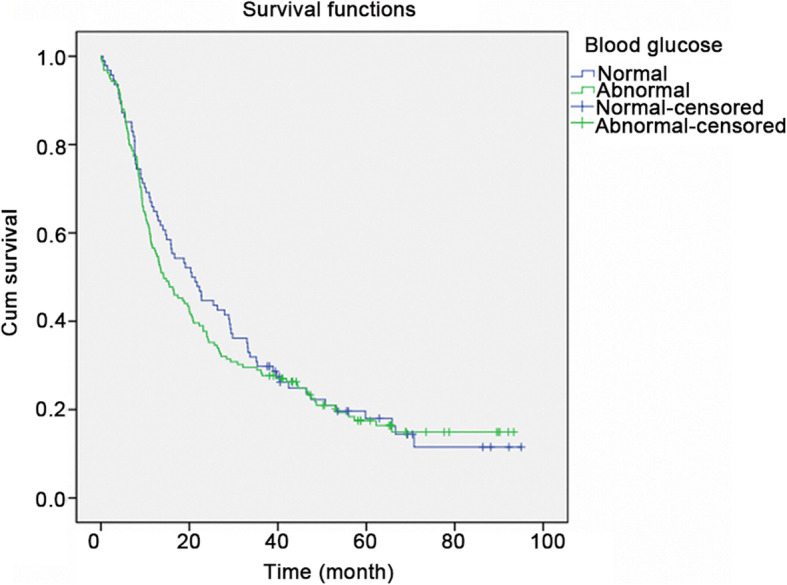
Kaplan-Meier survival curve. The median survival in the normal glucose group was 20.5 months (95% confidence interval (CI): 14.2–26.9). The median survival in the abnormal glucose group was 14.2 months (95% CI: 9.7–18.6). There was no statistically significant difference between the two groups (*P* = 0.573)

The relation between hyperglycemia and OS was also evaluated using a Kaplan-Meier analysis (Fig. 1). The median survival in the normal glucose group was 20.5 months (95% confidence interval (CI): 14.2–26.9). The median survival in the abnormal glucose group was 14.2 months (95% CI: 9.7–18.6). There was no statistically significant difference between the two groups (*P* = 0.573).

Univariable and multivariable logistic regression analyses were also performed for 1- and 2-year OS. No significant association was observed for glucose at 1-year OS (OR = 1.27, 95%CI: 0.71–2.29, *P* = 0.418); however, there was an association with CA199 > 1000 (OR = 3.68, 95%CI: 1.38–9.81, *P* = 0.009), moderate/high differentiation (OR = 0.51, 95%CI: 0.26–0.97, *P* = 0.039), and AJCC8 stage III-IV (OR = 2.51, 95%CI: 1.15–5.51, *P* = 0.021) (Table 3). There was also no significant association of glucose with 2-year OS (HR = 1.37, 95%CI: 0.76–2.46, *P* = 0.296); however, at this time point CA199 37–100 (OR = 2.46, 95%CI: 1.25–4.86, *P* = 0.009), CA199 > 1000 (OR = 3.33, 95%CI: 1.27–8.71, *P* = 0.014), and tail location (OR = 2.57, 95%CI: 1.19–5.53, *P* = 0.016) were significantly associated (Table 4).

**Table 3:** Univariable and multivariable logistic regression analyses of 1-year OS

	Univariable			Multivariable		
	OR	95% CI	P	OR	95% CI	P
Age	1.03	(1,1.06)	0.037	1.02	(0.99,1.06)	0.157
BMI	0.95	(0.88,1.03)	0.232			
Blood glucose						
Normal						
Abnormal	1.42	(0.84,2.4)	0.195	1.27	(0.71,2.29)	0.418
Sex						
Female						
Male	1.44	(0.86,2.41)	0.166			
Smoking history						
No						
Yes	0.9	(0.53,1.52)	0.691			
Drinking history						
No						
Yes	0.92	(0.48,1.74)	0.787			
Diabetes						
No						
Yes	1.5	(0.85,2.65)	0.161			
Hypertension						
No						
Yes	0.9	(0.51,1.59)	0.726			
Allergic history						
No						
Yes	0.96	(0.45,2.02)	0.908			
Abdominal pain						
No						
Yes	0.84	(0.49,1.46)	0.538			
Back pain						
No						
Yes	0.49	(0.23,1.06)	0.070	0.45	(0.18,1.11)	0.085
Anorexia						
No						
Yes	1.01	(0.28,3.68)	0.987			
Loss of appetite						
No						
Yes	1.49	(0.89,2.49)	0.126			
Loss of weight						
No						
Yes	0.8	(0.47,1.34)	0.390			
CA199						
Normal						
37–1000	1.82	(0.91,3.66)	0.092	1.7	(0.8,3.63)	0.171
> 1000	3.96	(1.59,9.89)	0.003	3.68	(1.38,9.81)	0.009
Procedure						
Whipple						
Distal	1.10	(0.59,2.04)	0.774			
Other	0.75	(0.18,3.08)	0.686			
Location						
Head						
Tail	1.17	(0.63,2.18)	0.618			
Differentiation						
Low						
Moderate-high	0.48	(0.27,0.85)	0.013	0.51	(0.26,0.97)	0.039
AJCC8 staging						
Stage I						
Stage II	1.54	(0.88,2.73)	0.134	1.54	(0.81,2.92)	0.185
Stage III-IV	1.95	(0.98,3.89)	0.058	2.51	(1.15,5.51)	0.021
Obstructive jaundice						
No						
Yes	1.07	(0.65,1.76)	0.799			

*OR* odds ratio, *CI* confidence interval, *BMI* body mass index, *CA* cancer antigen, *AJCC* American Joint Committee on Cancer

**Table 4:** Univariable and multivariable logistic regression analyses of 2-year OS

	Univariable			Multivariable		
	OR	95% CI	P	OR	95% CI	P
Age	1.02	(0.99,1.05)	0.235			
BMI	0.97	(0.89,1.05)	0.424			
Blood glucose						
Normal						
Abnormal	1.41	(0.84,2.36)	0.198	1.37	(0.76,2.46)	0.296
Sex						
Female						
Male	1.11	(0.66,1.85)	0.696			
Smoking history						
No						
Yes	1.22	(0.72,2.06)	0.462			
Drinking history						
No						
Yes	1.28	(0.66,2.45)	0.464			
Diabetes						
No						
Yes	1.67	(0.91,3.04)	0.096	1.5	(0.74,3.01)	0.259
Hypertension						
No						
Yes	0.9	(0.51,1.57)	0.702			
Allergic history						
No						
Yes	1.59	(0.72,3.51)	0.248			
Abdominal pain						
No						
Yes	1.5	(0.87,2.6)	0.145			
Back pain						
No						
Yes	0.61	(0.31,1.22)	0.164			
Anorexia						
No						
Yes	0.65	(0.18,2.3)	0.502			
Loss of appetite						
No						
Yes	1.24	(0.74,2.08)	0.410			
Loss of weight						
No						
Yes	0.76	(0.45,1.29)	0.315			
CA199						
Normal						
37–1000	2.1	(1.1,4)	0.025	2.46	(1.25,4.86)	0.009
> 1000	3.04	(1.22,7.62)	0.017	3.33	(1.27,8.71)	0.014
Procedure						
Whipple						
Distal	1.64	(0.85,3.16)	0.138			
Other	1.46	(0.35,6.01)	0.601			
Location						
Head						
Tail	1.76	(0.91,3.42)	0.096	2.57	(1.19,5.53)	0.016
Differentiation						
Low						
Moderate-high	0.62	(0.34,1.14)	0.126			
AJCC8 staging						
Stage I						
Stage II	0.93	(0.53,1.63)	0.807			
Stage III-IV	1.79	(0.86,3.73)	0.122			
Obstructive jaundice						
No						
Yes	0.82	(0.49,1.36)	0.439			

*OR* odds ratio, *CI* confidence interval, *BMI* body mass index, *CA* cancer antigen, *AJCC* American Joint Committee on Cancer

## Discussion

The association of fasting blood glucose with staging and overall survival of pancreatic cancer patients is still controversial [14, 15]. Therefore, this study aimed to investigate the association between fasting blood glucose levels and the OS of patients with pancreatic cancer and to evaluate the impact of differentiation and staging of pancreatic cancer. The results strongly suggest that fasting blood glucose levels are not associated with the OS of patients with pancreatic adenocarcinoma. Cancer differentiation degree and staging of pancreatic cancer did not influence the results for OS except at 1-year when they were both independently associated with OS.

In the present study, hyperglycemia and glucose levels were not associated with the OS of pancreatic cancer. This result suggests that pancreatic cancer is different to other solid tumors in general where hyperglycemia and diabetes are associated with prognosis [10–12]. The main mechanism involves insulin and insulin growth factor (IGF), which stimulate tumor growth [17–19]. The chronic inflammation state associated with hyperglycemia and diabetes also contributes to cancer progression [20]. However, the results of this study are supported by Dehayem et al. [21], who showed no association between diabetes and the prognosis of pancreatic cancer. Busaidy et al. [22] and Olowokure et al. [23] even showed that patients with pancreatic cancer and diabetes had a better OS than those without diabetes. On the other hand, some previous studies reported that hyperglycemia or diabetes was associated with poor survival of pancreatic cancer [24–28]. Those discrepancies and conflicting conclusions could be due to the sample size, ethnicity, and the fact that mortality can be due to diabetic complications rather than to cancer. In this study, we also considered that the degree of cancer differentiation or cancer stage might influence the results. So, we investigated whether these factors were associated with OS. The results suggest that for OS neither degree of differentiation nor cancer stage were associated factors. However, we did find that they were independently associated with 1-year OS. This might suggest that the results of other studies have been influenced by the differentiation degree or cancer stage of the patients, especially if the follow-up was only for 1 year. Larger case-control studies might be needed to fully evaluate the influence of these factors on OS.

The only factor in this study that was independently associated with the OS to pancreatic cancer was CA199. This is supported by previous studies that showed that CA199 levels are associated with the survival and response to chemotherapy in patients with pancreatic cancer [29–31].

This study has limitations. This was a cross-sectional study, which precludes the determination of causality relationships. In addition, the sample size was small and was from a single center, which could introduce bias. Because this was a retrospective study, only the information found in the charts could be analyzed.

## Conclusions

In conclusion, fasting blood glucose levels are not associated with the OS of patients with pancreatic adenocarcinoma. Only the CA199 levels are associated with OS. The results need to be confirmed using large-scale studies.

## Data Availability

Data sharing is not applicable to this article as no datasets were generated or analyzed during the current study.

## Abbreviations

OS: Overall survival
CI: Confidence interval
CT: Computed tomography
MRI: Magnetic resonance imaging
BMI: Body mass index
IGF: Insulin growth factor
